# Supplementary material for: Study on the Source and Microbial Mechanisms Influencing Heavy Metals and Nutrients in a Subtropical Deep-Water Reservoir
Source: Microorganisms. 2025 Dec 3;13(12):2750. doi: 10.3390/microorganisms13122750 (PMC12735212; doi:10.3390/microorganisms13122750)
Supplement: Supplementary file 1 [file microorganisms-13-02750-s001.zip › microorganisms-3911487-supplementary.pdf]

Supplementary Materials for:

Study on the Source and Microbial Mechanisms Influencing Heavy Metals and Nutrients in a Subtropical Deep-Water Reservoir

Gaoyang Cui [1,2,3], Jiaoyan Cui [1,2], Mengke Zhang [1,2], Boning Zhang [1,2], Yingying Huang [1,2], Yiheng Wang [1,2], Wanfu Feng [4,5], Jiliang Zhou [4,5], Yong Liu [4,5] and Tao Li [1,2,3,*]

[1] Faculty of Geographical Science and Engineering, College of Geographical Science, Henan University, Zhengzhou 450046, China
[2] Henan Dabieshan National Field Observation and Research Station of Forest Ecosystem, Zhengzhou 450046, China
[3] Xinyang Academy of Ecological Research, Xinyang 464000, China
[4] The Forest Science Research Institute of Xinyang, Xinyang 464031, China
[5] Henan Jigongshan Forest Ecosystem National Observation and Research Station, Xinyang 464031, China
* Correspondence: taodeny@163.com

Editing Certificate:

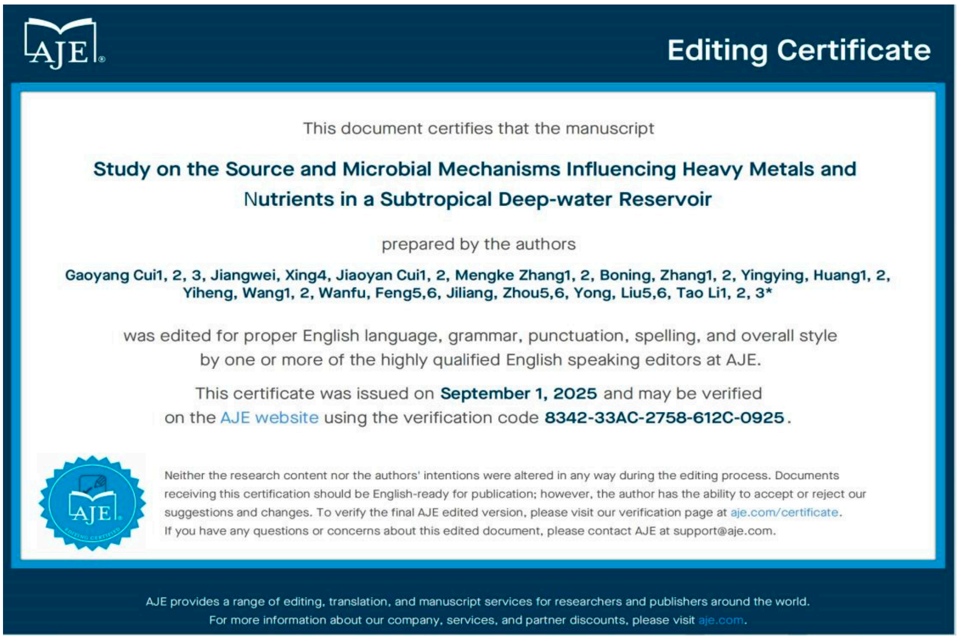
AJE

Editing Certificate

This document certifies that the manuscript

Study on the Source and Microbial Mechanisms Influencing Heavy Metals and Nutrients in a Subtropical Deep-water Reservoir

prepared by the authors

Gaoyang Cui1, 2, 3, Jiangwei, Xing4, Jiaoyan Cui1, 2, Mengke Zhang1, 2, Boning, Zhang1, 2, Yingying, Huang1, 2, Yiheng, Wang1, 2, Wanfu, Feng5,6, Jiliang, Zhou5,6, Yong, Liu5,6, Tao Li1, 2, 3*

was edited for proper English language, grammar, punctuation, spelling, and overall style by one or more of the highly qualified English speaking editors at AJE.

This certificate was issued on **September 1, 2025** and may be verified on the AJE website using the verification code **8342-33AC-2758-612C-0925**.

Neither the research content nor the authors' intentions were altered in any way during the editing process. Documents receiving this certification should be English-ready for publication; however, the author has the ability to accept or reject our suggestions and changes. To verify the final AJE edited version, please visit our verification page at aje.com/certificate. If you have any questions or concerns about this edited document, please contact AJE at support@aje.com.

AJE provides a range of editing, translation, and manuscript services for researchers and publishers around the world. For more information about our company, services, and partner discounts, please visit aje.com.

Academic Editor: Carlos A. Jerez

Received: 19 September 2025
Revised: 11 November 2025
Accepted: 27 November 2025
Published: 2 December 2025

Citation: Cui, G.; Cui, J.; Zhang, M.; Zhang, B.; Huang, Y.; Wang, Y.; Feng, W.; Zhou, J.; et al. Impact of Heavy Metals and Nutrients on Microbial Community Composition in a Subtropical Deep-Water Reservoir. *Microorganisms* **2025**, *13*, x. https://doi.org/10.3390/xxxxx

Copyright: © 2025 by the authors. Submitted for possible open access publication under the terms and conditions of the Creative Commons Attribution (CC BY) license (https://creativecommons.org/licenses/by/4.0/).

Captions:

Figure S1 Stratum clustering analysis of samples in four season

Figure S2 Heatmap of community composition at the genus level

Figure S3 Heatmap of the distribution of SOB/SRB annotated with the *aprAB* and *dsrAB*

Figure S4 Heatmap of the distribution of denitrifying bacteria annotated with the *nosZ* gene

Table S1 Concentration ranges of nutrients in the study.

Table S2 KEGG numbers for key steps in sulfur metabolism and annotation information

Table S3 KEGG numbers for key steps in nitrogen metabolism and annotation information

Table S4 Concentration ranges of dissolved heavy metals in the study

Table S5 Variation range of basic physical and chemical parameters of study area

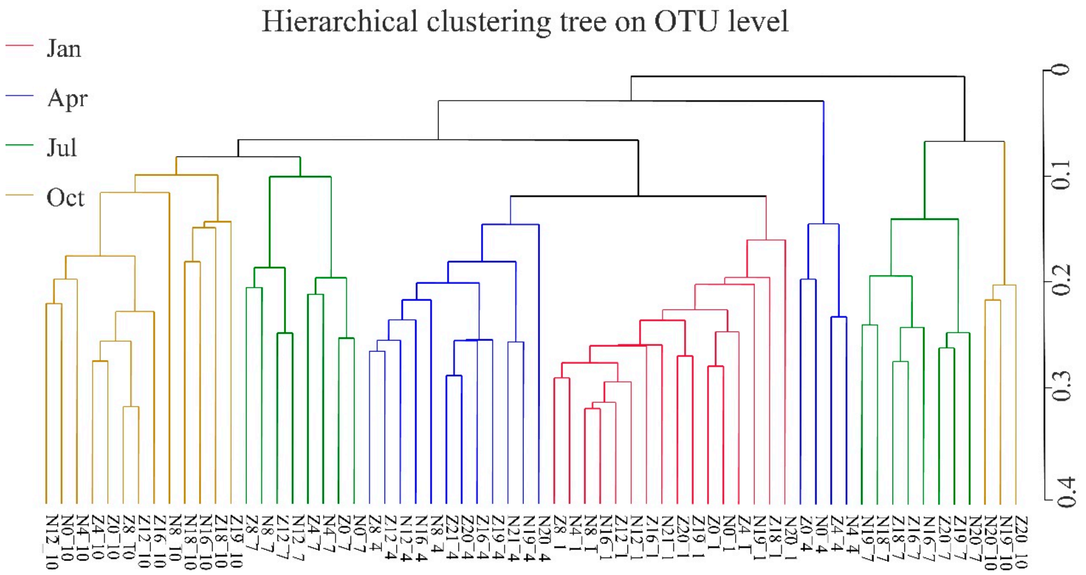

Figure S1 Stratum clustering analysis of samples in four season

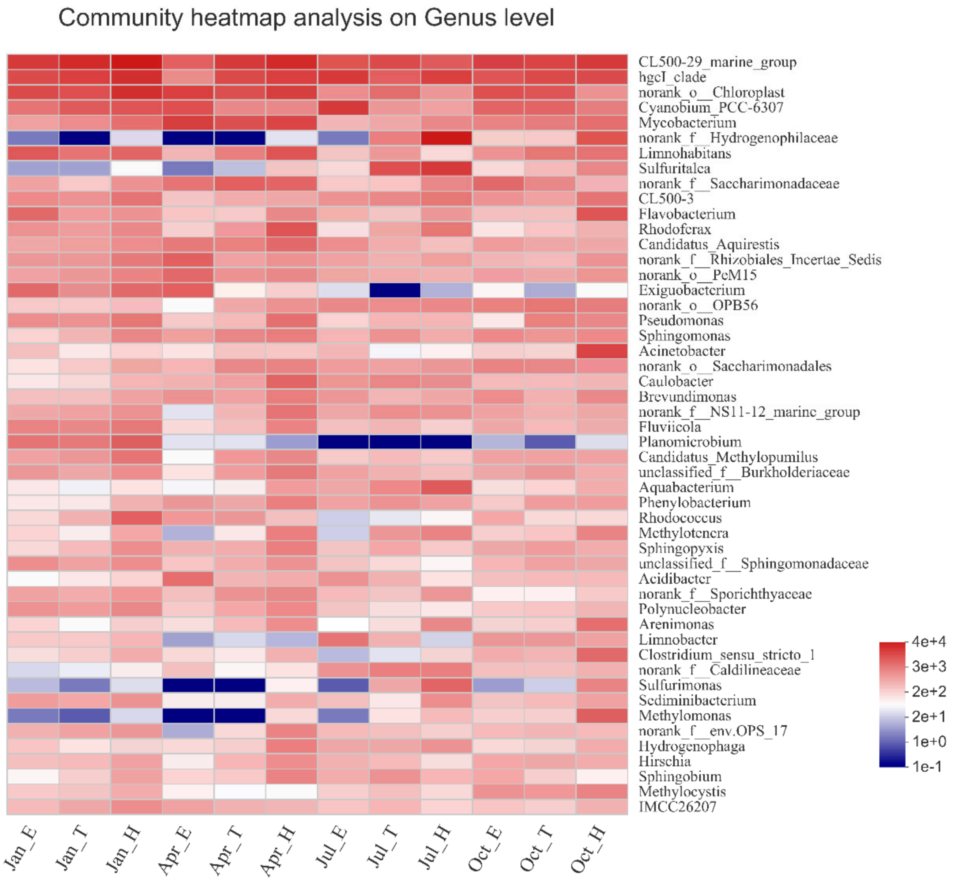

Figure S2 Heatmap of community composition at the genus level

Heatmap analysis of ***aprAB*** and ***dsrAB*** annotation

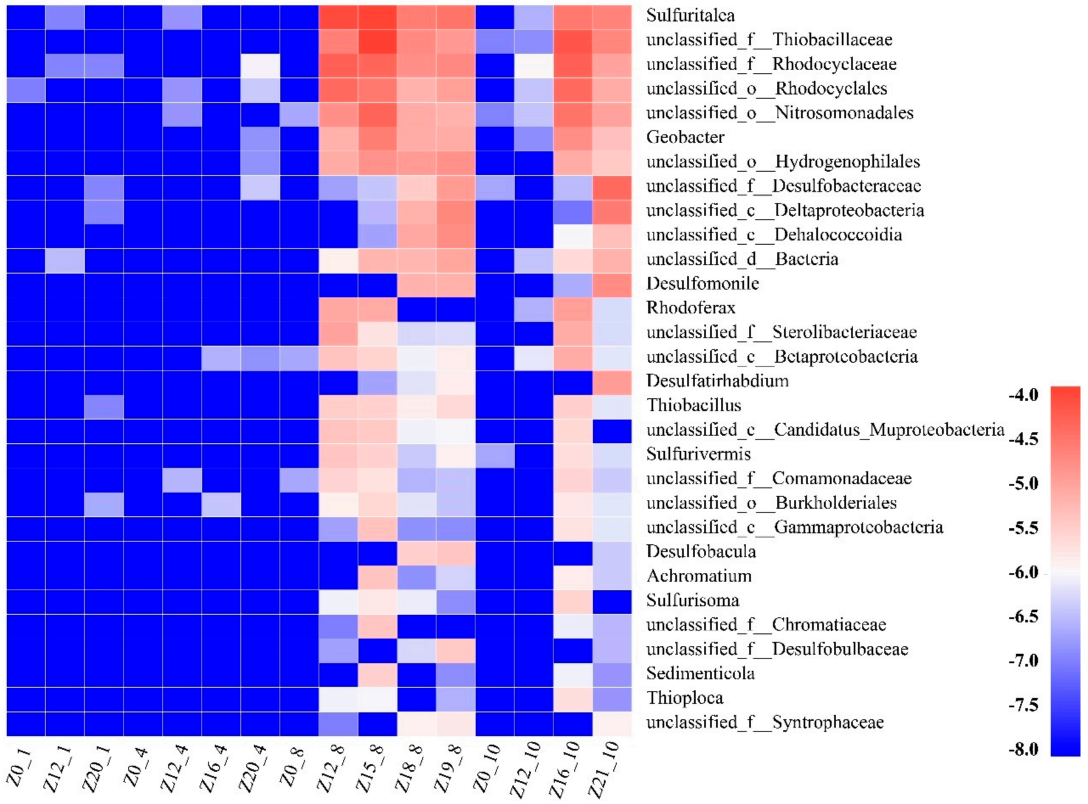

Figure S3 Heatmap of the distribution of SOB/SRB annotated with the *aprAB* and *dsrAB*

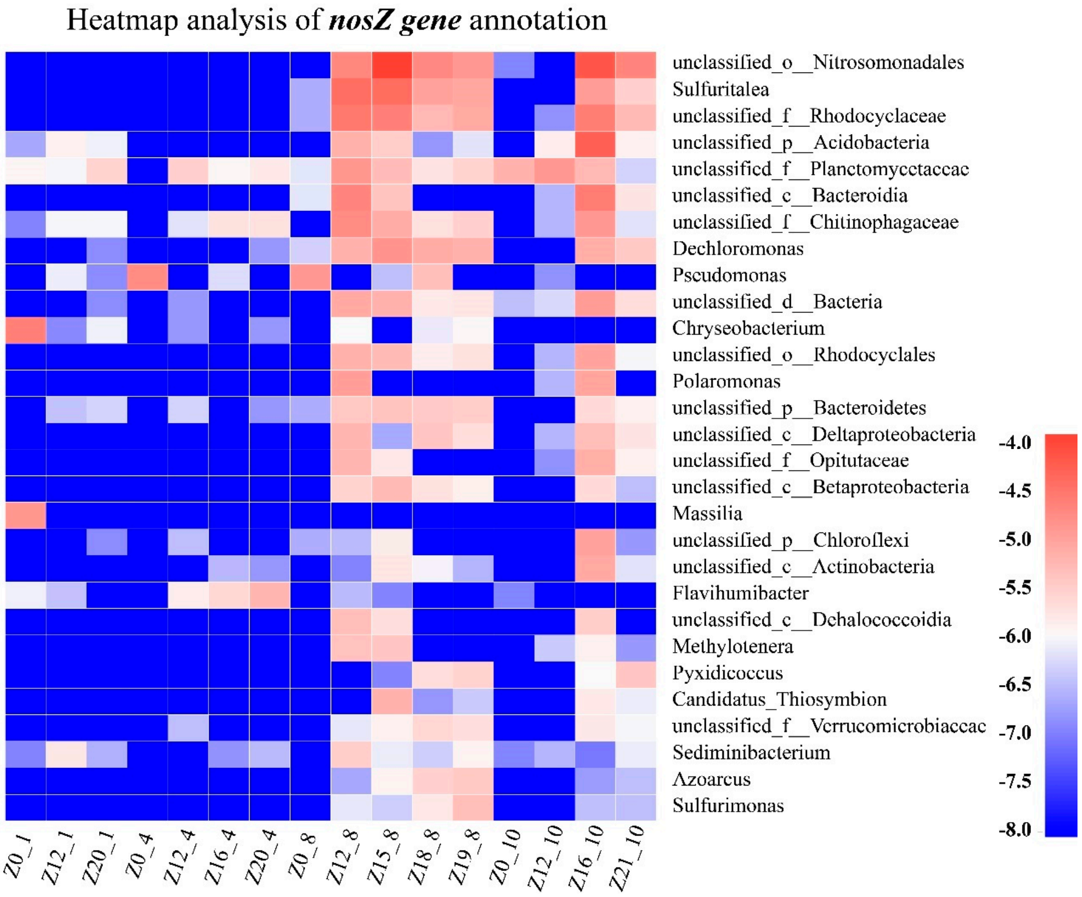

Figure S4 Heatmap of the distribution of denitrifying bacteria annotated with the *nosZ* gene

Table S1 Concentration ranges of nutrients in the study

Time		TDN (mg·L^{-1})	$NO3^-$-N (mg·L^{-1})	$NO2^-$-N (mg·L^{-1})	NH_4^+-N (mg·L^{-1})	DON (mg·L^{-1})	TDP (mg·L^{-1})	PO_4^{3-}-P (mg·L^{-1})	DSi (mg·L^{-1})
Jan	Content	3.15-11.9	1.66-7.04	0-0.16	0-0.50	1.52-4.95	0.14-0.50	0.01-0.32	2.73-7.28
	Average	5.44	3.13	0.02	0.04	2.26	0.25	0.11	3.62
Apr	Content	2.24-10.7	1.99-10.7	0.00-0.05	0.00-0.15	0.00-0.35	0.00-0.18	0.00-0.14	0.31-4.53
	Average	5.44	3.92	0.02	0.05	0.07	0.19	0.10	3.13
Jul	Content	2.10-6.92	1.38-6.37	0.00-0.11	0.00-0.66	0.00-0.48	0.00-0.19	0.00-0.17	0.21-5.19
	Average	3.58	3.20	0.04	0.16	0.17	0.07	0.07	2.15
Oct	Content	4.49-6.82	1.99-3.10	0.00-0.09	0.00-0.10	2.33-3.76	0.11-0.31	0.06-0.25	1.72-6.18
	Average	5.43	2.35	0.03	0.00	3.03	0.20	0.14	3.63

Table S2 KEGG numbers for key steps in sulfur metabolism and annotation information

Step	**KEGG**	**Gene**
Assimilatory sulfate reduction, sulfate => H_2S	K13811	PAPSS; 3'-phosphoadenosine 5'-phosphosulfate synthase
	K00958	sat, met3; sulfate adenylyltransferase
	K00955	cysNC; bifunctional enzyme CysN/CysC
	K00957	cysD; sulfate adenylyltransferase subunit 2
	K00956	cysN; sulfate adenylyltransferase subunit 1
	K13811	PAPSS; 3'-phosphoadenosine 5'-phosphosulfate synthase
	K00860	cysC; adenylylsulfate kinase
	K00955	cysNC; bifunctional enzyme CysN/CysC
	K00390	cysH; phosphoadenosine phosphosulfate reductase
	K00380	cysJ; sulfite reductase （NADPH） flavoprotein alpha-component
	K00381	cysI; sulfite reductase （NADPH） hemoprotein beta-component
	K00392	sir; sulfite reductase （ferredoxin）
Dissimilatory sulfate reduction, sulfate => H_2S	K00958	sat, met3; sulfate adenylyltransferase
	K00394	aprA; adenylylsulfate reductase, subunit A
	K00395	aprB; adenylylsulfate reductase, subunit B
	K11180	dsrA; dissimilatory sulfite reductase alpha subunit
	K11181	dsrB; dissimilatory sulfite reductase beta subunit
Thiosulfate oxidation by SOX complex	K17222	soxA; L-cysteine S-thiosulfotransferase
	K17223	soxX; L-cysteine S-thiosulfotransferase
	K17224	soxB; S-sulfosulfanyl-L-cysteine sulfohydrolase
	K17225/K22622	soxC/soxD; sulfane dehydrogenase subunit SoxC/soxD
	K17226	soxY; sulfur-oxidizing protein SoxY
	K17227	soxZ; sulfur-oxidizing protein SoxZ
Sulfate-sulfur assimilation	K02048/K23163	cysP; sulfate transport system substrate-binding protein
	K02046	cysU; sulfate transport system permease protein
	K02047	cysW; sulfate transport system permease protein
	K02045	cysA; sulfate transport system ATP-binding protein

Table S3 KEGG numbers for key steps in nitrogen metabolism and annotation information

Step	KEGG	Gene
Denitrification, nitrate => nitrogen	K00370	（narG, narZ, nxrA）; nitrate reductase / nitrite oxidoreductase, alpha subunit
	K00371	（narH, narY, nxrB）; nitrate reductase / nitrite oxidoreductase, beta subunit
	K00374	（narI, narV）; nitrate reductase gamma subunit
	K02567	（napA）; nitrate reductase (cytochrome)
	K02568	（napB）; nitrate reductase (cytochrome), electron transfer subunit
	K00368	（nirK）; nitrite reductase (NO-forming)
	K15864	（nirS）; nitrite reductase (NO-forming) / hydroxylamine reductase
	K04561	（norB）; nitric oxide reductase subunit B
	K02305	（norC）; nitric oxide reductase subunit C
	K00376	（nosZ）; nitrous-oxide reductase
Nitrogen fixation, nitrogen => ammonia	K02588	nifH; nitrogenase iron protein NifH
	K02586	nifD; nitrogenase molybdenum-iron protein alpha chain
	K02591	nifK; nitrogenase molybdenum-iron protein beta chain
	K00531	anfG; nitrogenase delta subunit
Assimilatory nitrate reduction, nitrate => ammonia	K00367	narB; ferredoxin-nitrate reductase
	K10534	NR; nitrate reductase (NAD(P)H)
	K00372	nasA; assimilatory nitrate reductase catalytic subunit
	K00360	nasB; assimilatory nitrate reductase electron transfer subunit
	K00366	nirA; ferredoxin-nitrite reductase
Dissimilatory nitrate reduction, nitrate => ammonia	K00370	narG, narZ, nxrA; nitrate reductase / nitrite oxidoreductase, alpha subunit
	K00371	narH, narY, nxrB; nitrate reductase / nitrite oxidoreductase, beta subunit
	K00374	narI, narV; nitrate reductase gamma subunit
	K02567	napA; nitrate reductase (cytochrome)
	K02568	napB; nitrate reductase (cytochrome), electron transfer subunit
	K00362	nirB; nitrite reductase (NADH) large subunit
	K00363	nirD; nitrite reductase (NADH) small subunit
	K03385	nrfA; nitrite reductase (cytochrome c-552)

	K15876	nrfH; cytochrome c nitrite reductase small subunit
	K10944	pmoA-amoA; methane/ammonia monooxygenase subunit A
Nitrification, ammonia => nitrite	K10945	pmoB-amoB; methane/ammonia monooxygenase subunit B
	K10946	pmoC-amoC; methane/ammonia monooxygenase subunit C
	K10535	hao; hydroxylamine dehydrogenase

Table S4 Concentration ranges of dissolved heavy metals in the study

Time		Cr ($\mu g \cdot L^{-1}$)	Mn ($\mu g \cdot L^{-1}$)	Fe ($\mu g \cdot L^{-1}$)	Co ($\mu g \cdot L^{-1}$)	Ni ($\mu g \cdot L^{-1}$)	Cu ($\mu g \cdot L^{-1}$)	Zn ($\mu g \cdot L^{-1}$)	As ($\mu g \cdot L^{-1}$)	Cd ($\mu g \cdot L^{-1}$)	Pb ($\mu g \cdot L^{-1}$)
Jan	Content	0.34-1.8	0.31-12.2	23.67-50.27	0.04-1.4	0.68-2.92	0.41-1.48	1.11-84.69	1.20-9.41	0.01-0.1	0.02-0.48
	Average	0.79	1.56	30.74	0.13	1.02	0.67	12.69	1.90	0.02	0.14
Apr	Content	0.50-0.9	0.27-27.8	17.34-38.48	0.05-1.7	0.38-3.06	0.42-0.78	0.69-20.18	1.03-8.42	0.00-0.4	0.02-0.14
	Average	0.73	2.79	27.60	0.15	1.00	0.50	4.96	1.74	0.04	0.05
Jul	Content	0.19-2.6	0.22-15.1	12.66-42.84	0.05-2.3	0.35-3.65	0.36-1.05	1.03-62.41	0.94-7.32	0.00-0.0	0.02-1.74
	Average	0.65	1.97	27.30	0.20	1.16	0.58	18.85	1.52	0.02	0.19
Oct	Content	0.18-1.2	0.19-13.7	23.48-35.60	0.05-2.3	0.70-2.73	0.42-0.90	0.70-13.01	1.30-8.48	0.01-0.0	0.01-0.09
	Average	0.30	1.32	26.84	0.18	0.92	0.61	3.65	1.91	0.02	0.04

Table S5 Variation range of basic physical and chemical parameters of study area

name	value	T(℃)	pH	ORP(mV)	DO(mg·L^{-1})	Chl（μg·L^{-1}）
Yangshui River	average	10.01	7.94	193.7	9.46	3.45
	range	9.43～10.51	7.87～	178.0～	9.22～9.92	0.38～6.25
Xiangjiang River	average	17.75	8.07	--	8.03	2.17
	range	15.68～	7.83～	--	7.71～8.35	0.76～3.20
Datang River	average	23.88	8.14	88.63	7.33	1.79
	range	21.51～	7.85～	72.00～	7.02～7.55	0.61～3.32
Wengan River	average	17.47	8.04	131.8	8.42	0.12
	range	15.76～	7.84～	115.1～	8.15～8.56	0.00～0.36
